# Notes of the Week

**Published:** 1921-07-16

**Authors:** 


### NOTES OF THE WEEK.

### VOLUNTARY HOSPITALS COMMISSION.

The Central Voluntary Hospitals Commission, recommended by Lord Cave's Committee, has now been appointed by the Minister of Health, and the members nominated by him are:—The Earl of Onslow, (Chairman), Lord Clwyd, Captain W. E. Elliott, M.C., M.P., Sir Robert Hudson, G.B.E., and Mr. D. O. Malcolm. The Marquis of Linlithgow is nominated by the Secretary for Scotland, Sir Napier Burnett by the British Red Cross Society, Sir Cooper Perry by King Edward's Hospital Fund, Mr. H. Wade Deacon by the British Hospitals Association, Sir John Rose Bradford by the Royal College of Physicians, Sir George Makins by the Royal College of Surgeons, and Dr. R. A. Bolam by the British Medical Association. The remaining member is to be nominated by the Scottish Committee of the British Medical Association. We have here a small but very useful body of men who are thoroughly versed in public affairs and many of whom have an intimate knowledge of voluntary hospital work; although detail in any degree does not come within their province, their qualifications cannot fail to be useful in dealing with the large matters of policy connected with the work of voluntary hospitals in this country. We are glad to see that Mr. L. G. Brock, C.B., has been appointed by the Minister of Health as Secretary to the Commission. Mr. Brock's work in connection with the National Relief Fund and Lord Cave's Committee is too recent and well known for further words of appreciation here, and his appointment ensures that the work of the secretariat will be carried out with complete efficiency.

### MEDICAL MEN AT NEWCASTLE.

The eighty-ninth annual meeting of the British Medical Association, which begins this week at Newcastle-on-Tyne, will continue till July 23; the representative meetings and functions take place on the 15th, 16th, 18th, and 19th, whilst the sectional meetings and demonstrations will be held on the 20th, 21st, and 22nd. This part of the annual meeting is introduced by a United Religious Service, at 5 p.m., on July 19, at St. Nicholas Cathedral, and the President's address at 8 p.m. A special address by Sir Thomas Oliver on "Industrial Hygiene" will be given on July 22, at 8 p.m., and Sir Arthur Keith will deliver the popular lecture on Friday, July 22, the subject being "Evolutionary Wounds." Armstrong College and the buildings which adjoin it will be utilised for the purposes of the annual meeting, and the improved railway services should ensure a good attendance.

### THE VARIOUS TYPES OF ARTIFICIAL LIMBS.

The Minister of Pensions has appointed a Committee to inquire into the arrangements for the supply and repair of the various types of artificial limbs which are provided under the Royal Warrants and Orders in Council, and into the comparative advantages of the metal limb and the wooden limb; and to make recommendations thereon. The members of the Committee are:—The Right Hon. Sir Archibald Williamson, Bart., P.C., M.P. (Chairman); Professor A. F. C. Pollard; Major Maurice Sinclair, C.M.G., R.A.M.C.; Major M. P. Leahy; Major A. A. Atkinson; Sir Lisle Webb, K.B.E., C.B., C.M.G., Director-General of Medical Service, Ministry of Pensions; Mr. Frank Cecil Meech, ex-Corporal of Horse, Royal Horse Guards. Mr. W. J. Sullings has been appointed Secretary of the Committee. The inquiry should prove a useful one, and the report of the Committee will be awaited with interest.

### KING GEORGE'S SANATORIUM FOR SAILORS.

To the Seamen's Hospital Society belongs the credit of establishing the first sanatorium for seamen, and H.R.H. the Duke of York, in opening this institution on July 13, expressed his pleasure at this fact. King George's Sanatorium for Sailors, as it is called, has the further unique feature that cases of tubercle will be admitted in any stage of the disease, and that it is opened free of debt and with an endowment fund sufficient to enable it to carry on its work without help. The fund raised, thanks to Lord Inchcape's and Capt. Hodgkinson's advocacy, amounts to over £100,000; the Ministry of Health has given £14,500 towards the building of cubicles; King George's Fund, £6,950; and generous assistance has come from other bodies and funds. The sanatorium, which is situated at Bramshott, within a mile of Liphook station, is on an estate of 100 acres, and the first pavilions, now ready, provide for eighty beds on two floors. As a memorial to the valour of British seamen it must command the interest and sympathy of all, and H.R.H. at the opening ceremony said he knew no more fitting way to show thankfulness for victories at sea than by helping seamen in their hour of sickness.

### THE LATE SIR G. H. SAVAGE.

The profession has suffered a great loss with the death last week of Sir George Henry Savage, M.D., F.R.C.P., at the age of seventy-eight. Born at Brighton, though he came of a Yorkshire stock, he completed his medical training at Guy's, and, after holding house appointments at this hospital, went to Bethlem Royal Hospital, where he spent seventeen years. For seven he was assistant medical officer and for ten physician superintendent. At the time he first took up the psychological branch of medicine it was, though ardently followed on the Continent, still relatively neglected in this country. But later his teaching in his own wards and his classes at Guy's attracted many visitors from abroad, both under- and post-graduate. One of his earnest pleas was for more liberal State-aid towards scientific research into the prevention and cure of insanity. He felt it was necessary to consider the lunatic himself as much as society apart from the lunatic, and his views have nowadays many supporters. In 1889 he resigned his post at Bethlem, but continued to be a governor there. He was a consultant and adviser to the Government, an examiner to London University, and at various times president of many learned societies. His writings on his special subjects were extensive, and for a time he edited the *Journal of Mental Science*, but his most monumental work in this way was "A Manual of Insanity." He received his knighthood in 1912. Sir George married first, in 1867, a daughter of Mr. Jacob Walton, of Alston Moor, and second, in 1882, a daughter of Dr. Sutton, a physician to the London Hospital.

### ROYAL WESTMINSTER OPHTHALMIC HOSPITAL.

H.R.H. The Duke of Connaught, K.G., has signified his intention to visit this hospital on Monday next, July 18, at 3 o'clock.

### THE LATEST HOSPITAL OF RECOVERY.

Another link in the chain of development of the Great Northern Central Hospital was forged on Saturday, July 9, when its Hospital of Recovery at Grovelands, Southgate, was formally opened. The house at present accommodates forty-five patients, and there is room for extension to seventy. The men are in one ward, which is an annexe to the main building, and was used by military patients when the institution was the Southgate V.A.D. Hospital; the women are in three communicating wards on the ground floor, and the children's and babies' ward with six beds is on the first floor, where also there is an emergency operation-theatre. The house is a gift from the Southgate Voluntary-Aid Detachment. Surrounded by sixty acres of park-land, part of which is the Southgate Public Park, with ancient trees, a lake, and beautiful views, this hospital should prove an ideal home of recovery. The nursing staff consists of a matron, Miss Beatrice Everingham, a sister, two staff nurses, three assistant nurses, and four probationers, who after twelve months go to the mother hospital to complete their training. H.R.H. Princess Royal performed the opening ceremony, and a wonderful programme of sports, games, music, acting, and dancing was provided for the 16,000 visitors who in the most perfect of weather were finding entertainment and refreshment. The proceeds of the *fête* amounted to £1,100, a profitable result from three weeks' work. We hope this is an earnest of the interest that has arisen in this necessary addition to the service of a large urban general hospital, which was commented on and recommended in the report of Lord Cave's Committee. To maintain the various branches of the Great Northern Central—*i.e.*, the mother hospital, 210 beds, Grovelands, 45, the Convalescent Home, Clacton, 30, and the additional 81 to be provided under the amalgamation scheme with the Royal Chest Hospital—an annual income of £100,000 is required.

### MASS CONTRIBUTIONS.

Anything bearing upon the problem of organising mass contributions in London deserves consideration, and therefore we draw attention to a proposal which its author, Mr. G. Burnham, has offered to the British Hospitals Association, King Edward's Hospital Fund for London, and similar bodies. He proposes that an annual "King's Day" be held, if possible on His Majesty's birthday, and that on this national occasion *all* employers of labour, from the banker to the butcher, be invited to send to the hospital committee of their district (*a*) an annual donation, and (*b*) an undertaking to organise a system of weekly contributions from their staffs, the proceeds of which would also be sent to the local hospitals' committee. The author's suggestion is that the difficulties of organising mass contributions everywhere would be diminished if a national effort were made, and, further, that a national effort will have the greatest chance of success if made on one day and with the prestige of the King's name behind it. He argues, further, that the Cave Committee has recommended the formation of local hospital committees, for which his proposal is a supplementary effort, and that the idea of a "King's Day" would provide a short-cut to the organisation of the subscriptions needed. Even with the trumpet-call of such a "day," however, it is clear that immense work would fall upon the local committees if the bulk even of employers in their district were to be approached. The proposer, therefore, has offered his services as organiser to the King's Fund, being the body recommended by Lord Cave's Committee to represent the hospitals of the London area. Flag-days have raised large sums. Could a somewhat similar plan raise *annual* subscriptions and effective promises of the hard work involved by the introduction of weekly contributions in shops and offices hitherto ignorant of the system?

### A JOINT POLICY AT BRISTOL AND SHEFFIELD.

Although amalgamation has not taken place at Bristol, the General Hospital and the Royal Infirmary have been considering a common scheme whereby the system of contributions from patients may be the same at each institution. Before drafting the scheme, which began on July 1, inquiries were made of the results of similar efforts. The evidence, which came both from London and the provinces, is worth summarising. Mr. T. W. Gregg, the secretary of the Bristol General Hospital, reported that the contributions to the Metropolitan Hospital Saturday Fund have increased since patients' payments were started at the London hospitals, by which King's College is receiving £8,000 a year, and St. Mary's £180 a week. At Norwich, at Exeter and at Plymouth the scheme has also worked well. These few instances are sufficiently scattered to show that there is no type of district in which such a scheme may not be considered. In a few its adoption may prove inadvisable, but the remarkable fact is that, generally speaking, the plan has not affected any other sources of income. Joint action of a somewhat different kind is being taken by the Sheffield hospitals, where a revised system of recommendations under the Joint Hospitals Council has been adopted, the recommendations being available at any of the four Sheffield hospitals.

